# The Alkaloid Compound Harmane Increases the Lifespan of *Caenorhabditis elegans* during Bacterial Infection, by Modulating the Nematode’s Innate Immune Response

**DOI:** 10.1371/journal.pone.0060519

**Published:** 2013-03-27

**Authors:** Henrik Jakobsen, Martin S. Bojer, Martin G. Marinus, Tao Xu, Carsten Struve, Karen A. Krogfelt, Anders Løbner-Olesen

**Affiliations:** 1 Department of Science, Systems and Models, Roskilde University, Roskilde, Denmark; 2 Department of Microbiology and Infection Control, Statens Serum Institut, Copenhagen, Denmark; 3 Department of Biochemistry and Molecular Pharmacology, University of Massachusetts Medical School, Worcester, Massachusetts, United States of America; 4 Department of Biology, University of Copenhagen, Copenhagen, Denmark; The Scripps Research Institute and Sorrento Therapeutics, Inc., United States of America

## Abstract

The nematode *Caenorhabditis elegans* has in recent years been proven to be a powerful in vivo model for testing antimicrobial compounds. We report here that the alkaloid compound Harmane (2-methyl-β-carboline) increases the lifespan of nematodes infected with a human pathogen, the Shiga toxin-producing *Escherichia coli* O157:H7 strain EDL933 and several other bacterial pathogens. This was shown to be unrelated to the weak antibiotic effect of Harmane. Using GFP-expressing *E. coli* EDL933, we showed that Harmane does not lower the colonization burden in the nematodes. We also found that the expression of the putative immune effector gene *F35E12.5* was up-regulated in response to Harmane treatment. This indicates that Harmane stimulates the innate immune response of the nematode; thereby increasing its lifespan during bacterial infection. Expression of *F35E12.*5 is predominantly regulated through the p38 MAPK pathway; however, intriguingly the lifespan extension resulting from Harmane was higher in p38 MAPK-deficient nematodes. This indicates that Harmane has a complex effect on the innate immune system of *C. elegans*. Harmane could therefore be a useful tool in the further research into *C. elegans* immunity. Since the innate immunity of *C. elegans* has a high degree of evolutionary conservation, drugs such as Harmane could also be possible alternatives to classic antibiotics. The *C. elegans* model could prove to be useful for selection and development of such drugs.

## Introduction

For more than a decade now, the nematode *Caenorhabditis elegans* has been used as a simple infection model for several important human pathogens [1]. In recent years the nematode has also been used as an in vivo model to screen for compounds that combat these microbial infections [2], [3]. The nematode constitutes an attractive model since it allows identification of classic antibiotic compounds, as well as compounds that inhibit bacterial virulence or stimulate the nematode’s immune response. An example of such a compound, targeting the innate immune system of *C. elegans*, has recently been reported by Pukkila-Worley *et al.* [4]. They showed that the small molecule drug, named RPW-24, up-regulated several antimicrobial immune effector genes; resulting in increased lifespan of the worms, when infected with the pathogen *Pseudomonas aeruginosa*.

There are at least four pathways regulating immunity of *C. elegans*. These are the transforming growth factor-ß-like pathway, the p38 mitogen-activated protein kinase pathway (p38 MAPK pathway), the insulin-like receptor pathway, and the programmed cell death pathway [5]. Two of these are of particular interest, since they are known to promote longevity in *C. elegans* during infections of the intestine: the p38 MAPK pathway and the insulin-like receptor pathway. The p38 MAPK pathway has been shown to be induced in response to infection by specific human pathogens such as *P. aeruginosa* [6] and *Yersinia pestis* [7]. In contrast, the insulin-like receptor pathway is believed to provide the nematode with a continuous low-level protection against a broad range of pathogens [6], [8]. Apart from antimicrobial factors, these pathways also control the expression of proteins involved in protection against environmental stress.

Here we report that the alkaloid compound Harmane (2-methyl-β-carboline) increases the lifespan of *C. elegans* during infection by several pathogenic bacteria. Harmane was initially found as a hit in a compound library screen, using a bacterial two hybrid assay. In the screen we were trying to identify inhibitors of the interaction between the enterohemorrhagic *E. coli* virulence factors, Tir (translocated intimin receptor) and Intimin [9] (Method S1). We wanted to use the nematode model to verify the importance of the virulence factor and to show any in vivo efficacy of Harmane. We found that the virulence factor did not contribute to the pathogenicity of the bacteria against the nematode. However, we observed a marked increase in the lifespan of nematodes feeding on bacteria grown in the presence of Harmane (Figure S1). This led us to suspect that Harmane targeted an unknown virulence factor in the bacteria. Further studies, however, revealed that the target of Harmane was not in the bacteria, but in *C. elegans*. Based on our results we suggest that Harmane stimulates the immune/stress response in the nematode.

## Results and Discussion

### Harmane promotes longevity in C. elegans, infected with E. coli EDL933, in a dose dependent manner - unrelated to its antibiotic effect

**Figure 1:**
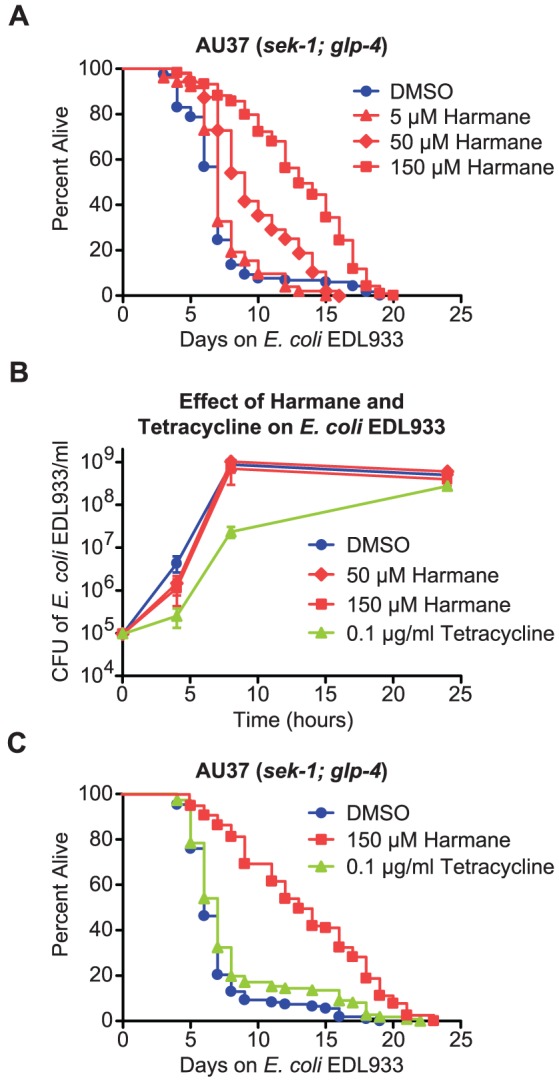
The effect of Harmane is dose-dependent, but unrelated to its antimicrobial effect. (A) *E. coli* EDL933 infection assay on *sek-1*; *glp-4* nematodes with different concentrations of Harmane compared to the solvent (DMSO). The lifespan extension is significant for the two highest concentrations of Harmane compared to DMSO (*P*<0.0001) [DMSO, n = 118; 5 µM, n = 52; 50 µM, n = 48; 150 µM, n = 119]. (B) Growth assay on *E. coli* EDL933 in NGM medium with different concentrations of Harmane or the solvent (DMSO), compared to *E. coli* EDL933 grown in NGM medium with 0.1 µg/ml of the antibiotic tetracycline. Both concentrations of Harmane affect the growth of EDL933, but not as much as 0.1 µg/ml of tetracycline. Data points are the average of three replicates and error bars represent SEM. (C) *E. coli* EDL933 infection assay on *sek-1*; *glp-4* nematodes with comparable concentrations of Harmane and tetracycline, compared to DMSO. Tetracycline, at 0.1 µg/ml, extends the lifespan significantly (*P* = 0.0283) as well does 150 µM Harmane (*P*<0.0001). However, the mean survival of nematodes on DMSO is 6 days, compared to 7 days for 0.1 µg/ml tetracycline and 13 days for 150 µM Harmane [DMSO, n = 108; 0.1 µg/ml Tetracycline, n = 111; 150 µM Harmane n = 117].

After discovering that Harmane had the ability to promote survival of C. elegans, infected with E. coli EDL933; we decided to investigate this effect more closely. We tested the effect of Harmane concentration on survival. This was performed in a standard agar-based infection assay, with an immuno-compromised mutant of C. elegans. This strain carries a mutation in the sek-1 gene of the p38 MAPK pathway, making it more susceptible to several pathogens [3], [10]. We found that the median survival of the nematodes changed from 7 days on the solvent DMSO or 5 µM Harmane, to 9 days on 50 µM Harmane and 13 days on 150 µM Harmane (Figure 1A). The lifespan of C. elegans did not change significantly at higher concentrations of Harmane (see Figure S1).We speculated whether this effect could be explained by a mere antimicrobial effect of Harmane. Harmane has previously been reported to have a minor antimicrobial effect against E. coli [11]. Reza et al. reported a minimum inhibitory concentration (MIC) of 0.6 mg/ml (3.29 mM). We found the MIC to be 0.5 mg/ml (2.74 mM) in nematode growth medium (NGM) (see Table S1 and Method S2). This was much higher than the highest concentration used in our assays, however, we decided to compare the antimicrobial effect of Harmane, to a comparable concentration of the traditional antibiotic tetracycline, in the survival assay. We grew E. coli EDL933 in triplicate in NGM medium, with different concentrations of Harmane or a low concentration of tetracycline, and plotted the number of colony forming units (CFU) against time (Figure 1B). Both Harmane and tetracycline had an inhibitory effect, on the growth of the bacteria. However, the inhibitory effect of 0.1 µg/ml tetracycline was stronger than that of both 50 and 150 µM of Harmane. We decided to compare the lifespan of C. elegans on plates with 0.1 µg/ml tetracycline and 150 µM of Harmane. We found that tetracycline had only a minor effect on the lifespan of the nematode, compared to Harmane (Figure 1C). This is similar to an earlier report, which found that tetracycline only rescued C. elegans from the pathogen Enterococcus faecalis, at concentrations several fold higher than the MIC [3]. We concluded that the lifespan extension from Harmane was not caused by its antimicrobial effect.

### Harmane extends the lifespan of sek-1 worms on several pathogenic bacteria as well as heat-killed E. coli EDL933

**Figure 2:**
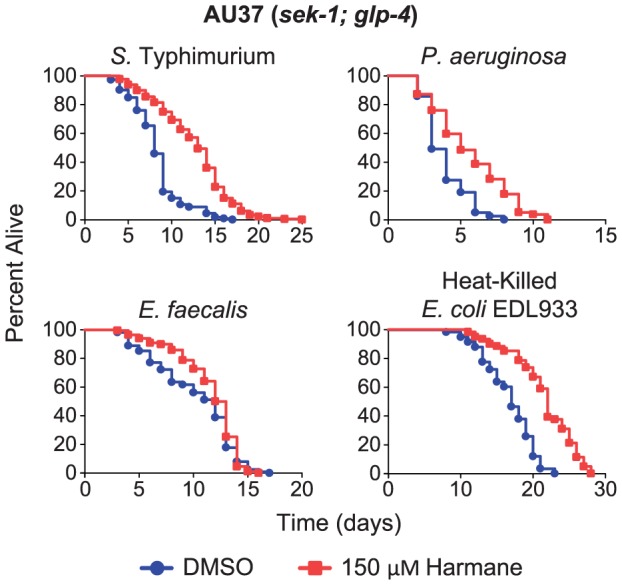
Harmane rescues *sek-1*; *glp-4* nematodes fed on different pathogens. There was a significant extension of life span of *C. elegans* feeding on bacteria grown on Harmane plates compared to plates with DMSO. The median survival when fed on *S*. Typhimurium was 8 days on DMSO and 13 days on 150 µM Harmane [*P*<0.0001; DMSO, n = 113; 150 µM Harmane, n = 180]. When fed on *P. aeruginosa* on DMSO, median survival was 3 days, and on 150 µM Harmane it was 5 days [*P*<0.0001; DMSO; n = 120; 150 µM Harmane, n = 134]. Feeding on *E. faecalis* on DMSO resulted in a median survival of 12 days, compared to 12.5 days on 150 µM Harmane [*P* = 0.0013; DMSO, n = 162; 150 µM Harmane, n = 198]. When feeding on heat-killed *E. coli* EDL933, the median survival was 17 days on DMSO, compared to 22 days on 150 µM Harmane [*P*<0.0001; DMSO, n = 58; 150 µM Harmane, n = 61].

At this point we still believed that the effect of Harmane was exerted on the bacteria, possibly by targeting an unknown virulence factor. In order to confirm this suspicion, we decided to test the ability of Harmane to rescue nematodes infected with other bacterial pathogens. We reasoned that if the target of Harmane was in the bacteria, it would be possible to find strains that lacked this target. We chose three pathogens known to be lethal to C. elegans: Salmonella serovar Typhimurium strain C17 [12], [13], Pseudomonas aeruginosa strain PA14 [14], and Enterococcus faecalis strain OG1RF [3], [15]. We also reasoned that if the target was indeed a virulence factor, we would see no life extending effect of Harmane, if nematodes were fed on dead bacteria. However, we found that Harmane was able to rescue the nematodes on all three pathogenic strains and to promote longevity even on heat-killed E. coli EDL933 (Figure 2). Taken together, these data indicated that the target of Harmane was in C. elegans, rather than in the pathogens. We had not noticed any avoidance behavior of the worms on plates with Harmane compared to the control plates with DMSO. However, we considered the possibility that Harmane could be toxic to the nematodes and alter their feeding behavior. We added Harmane or DMSO to the centre of lawns with non-pathogenic E. coli OP50. AU37 (sek-1; glp-4) nematodes were added to the lawns, and after 16 hours we scored the worms as either on the lawns or off the lawns (Figure S2 and Method S3). There was no significant difference between plates with Harmane and control plates (P = 0.2046). We concluded that Harmane was not toxic to C. elegans.

### Harmane does not reduce the colonization-burden of E. coli EDL933 in the nematode intestine

**Figure 3:**
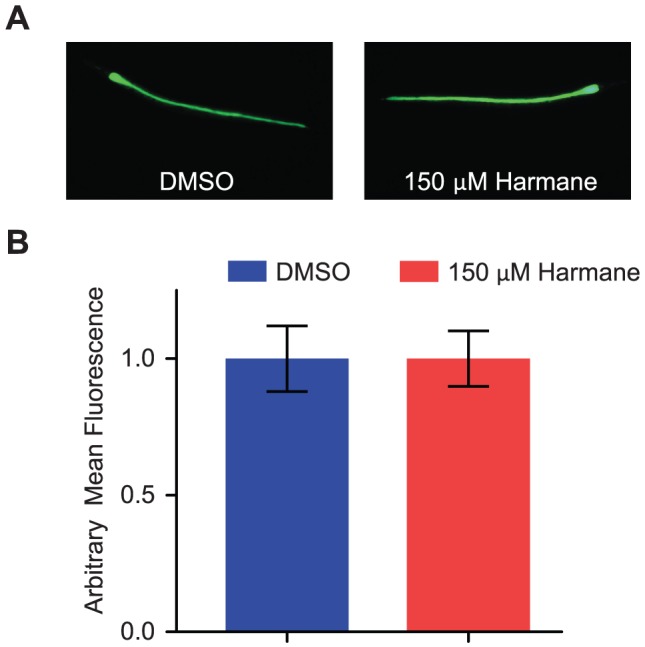
Harmane does not reduce the colonization-burden in *C. elegans* by *E. coli* EDL933. (A) Fluorescence microscopy pictures of *sek-1*; *glp-4* mutant *C. elegans* after feeding 4 days on GFP-expressing *E. coli* EDL933, grown on either DMSO or Harmane. The pictures show examples of strongly colonized individuals. (B) Individual nematodes in fluorescence pictures were quantified, and data normalized to the level of the DMSO treated nematodes. There was no significant difference between the two samples [DMSO, n = 56; 150 µM Harmane, n = 69; error bars indicate SEM].

We proceeded to determine whether Harmane caused a reduction in the colonization of the intestine of the nematode. A strong inverse correlation between the degree of bacterial colonization and the expected lifespan of the individual worms has recently been shown [16]. We expected that nematodes treated with Harmane would have an overall lower colonization-burden, compared to nematodes treated with DMSO. We used E. coli EDL933 carrying a plasmid expressing green fluorescent protein (GFP) [17], allowing us to visualize and quantify the bacteria inside the intestine (Figure 3A and 3B). Contrary to what we had expected, we found no difference in the colonization-burden, in worms treated with DMSO, and worms treated with Harmane. This prompted us to look for the Harmane target in the innate immune system of the nematode. Since all infection assays had been performed in the mutant nematode, with a defective p38 MAPK pathway; we reasoned that we should direct our attention towards the insulin-like receptor pathway.

### Harmane does not target the insulin-like receptor pathway of C. elegans

**Figure 4:**
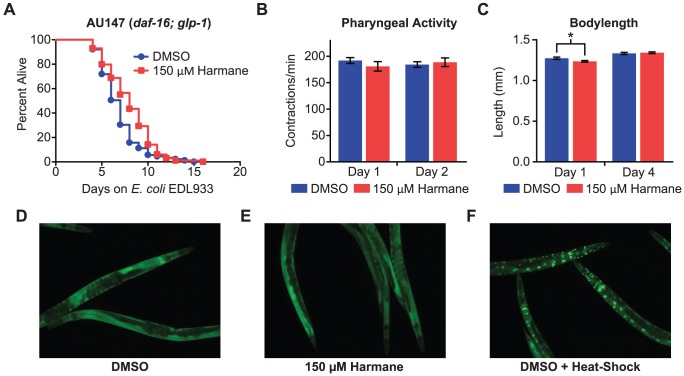
The effect of Harmane on the insulin-like receptor pathway. (A) *E. coli* EDL933 infection assay on *daf-16*; *glp-1* nematodes exposed to Harmane and DMSO. The median survival on Harmane was 8 days, compared to 7 days on DMSO. This was lower than the lifespan extension seen in *sek-1*; *glp-4* nematodes, however, still significantly different (*P*<0.0001) [DMSO, n = 89; 150 µM Harmane, n = 99]. (B) Pharyngeal pumping activity of *sek-1*; *glp-4* nematodes (AU37) fed on *E. coli* OP50, exposed to DMSO or Harmane [DMSO, n = 10; 150 µM Harmane, n = 10; error bars indicate SEM]. (C) Body length of *sek-1*; *glp-4* nematodes (AU37) fed on *E. coli* OP50, grown on DMSO or Harmane. Day 1 data shows a significant difference between Harmane and DMSO (*P* = 0.0242) [DMSO, day 1, n = 68; 150 µM Harmane, day 1, n = 72; DMSO, day 4, n = 72; 150 µM Harmane, day 4, n = 75; error bars indicate SEM]. (D) Fluorescence microscopy pictures of *daf-16::gfp* transgenic *C. elegans* after feeding 20 hours on *E. coli* OP50, grown on either DMSO or (E) 150 µSM Harmane. (F) Nematodes from a DMSO plate exposed to 37°C for 30 minutes, just prior to being analyzed (positive control of DAF-16 translocation to nucleus). We could not detect any effect of Harmane on DAF-16 in the worms, as a result of the Harmane treatment. Pictures taken after 1 hour exposure to DMSO or Harmane (not shown), were identical to the 20-hour pictures.

The insulin-like receptor pathway regulates the entry of C. elegans into the very long-lived dauer larval stage, instead of the normal L3 larval stage. The decision to enter the dauer stage must normally be made in the L1 larval stage. However, it has been shown that mutations in the daf-2 gene of the pathway also can cause fertile, active, adult nematodes to more than double their lifespan [18]. It is therefore believed that the insulin-like receptor pathway is responsible for regulating the nematodes basic response to environmental stress [8], [19]. This stress can be in the form of low food resources or pathogenic bacteria. Under such unfavorable conditions, the insulin-like receptor pathway is activated; as a result resources are diverged away from growth and reproduction, and towards stress resistance and longevity. The insulin-like receptor pathway controls the transcription of genes involved in stress resistance via the transcription factor DAF-16. The lifespan extending effect of daf-2 mutants has been shown to be completely suppressed by mutations in daf-16 [18]. We reasoned therefore, that if Harmane acted on the insulin-like receptor pathway, Harmane would have no effect on a daf-16; glp-1 mutant nematode. We tested the susceptibility of this strain to E. coli EDL933, with DMSO or 150 µM Harmane. We found that the effect of Harmane on the lifespan of daf-16; glp-1 mutants was considerably less pronounced, than in the sek-1; glp-4 mutant, although exposure to Harmane still resulted in significantly longer lifespan (Figure 4A). This indicated a possible interaction between the insulin-like receptor pathway and Harmane. In order to investigate this possible involvement of the insulin-like receptor pathway further, we assumed that activation of this pathway would also negatively affect traits such as, pharyngeal activity and body length of C. elegans. We performed the measurements of pharyngeal activity and body length on the sek-1; glp-4 mutant used in the first infection assay. We found no difference in the pharyngeal activity between nematodes exposed to Harmane compared to DMSO (Figure 4B). There was a small, but significant, difference in body length between nematodes exposed to Harmane for 1 day, compared to DMSO. However, such a difference could not be detected on day 4 (Figure 4C).

As mentioned above, activation of the insulin-like receptor pathway involves the transcription factor DAF-16. When not activated, DAF-16 is evenly distributed in the cytoplasm of all cells in the nematode. Upon activation DAF-16 is translocated from the cytoplasm to the nucleus. This translocation can be visualized using daf-16::gfp transgenic nematodes [19]. Transgenic nematodes were exposed to DMSO or Harmane for 1 hour or 20 hour, and then examined by fluorescence microscopy. As a positive control of DAF-16 translocation, we exposed the nematodes to 37°C for 30 minutes, just prior to examination. We detected no signs of DAF-16 translocation in response to Harmane (Figure 4D–E).

**Figure 5:**
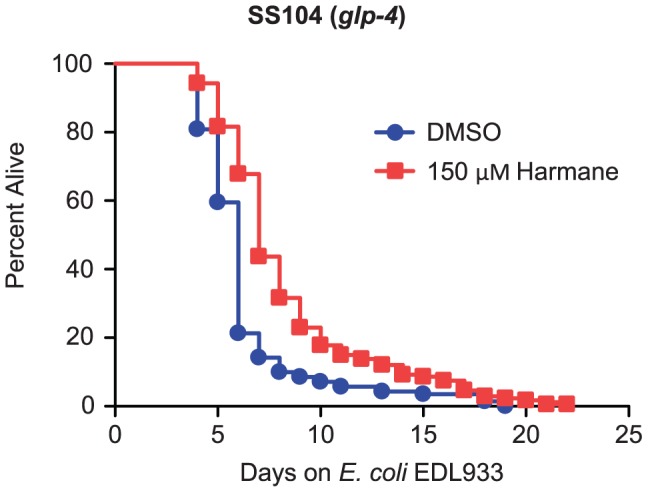
The effect of Harmane in *glp-4* nematodes. *E. coli* EDL933 infection assay with *glp-4* nematodes, exposed to DMSO or Harmane. The lifespan extension was from 6 days on DMSO to 7 days on Harmane [*P*<0.0001; DMSO, n = 141; 150 µM Harmane, n = 173].

These results were contradictory to our first observation, which showed that the daf-16; glp-1 mutant was less affected by Harmane. Hence, we decided to test the effect of Harmane on a glp-4 nematode. We reasoned that if the results were similar to the ones found with the sek-1; glp-4 mutant, we could confirm the insulin-like receptor pathway as a target for Harmane. We tested the effect of Harmane on a SS104 C. elegans strain (this carries the glp-4 mutation conferring temperature sensitive sterility, but has intact immune pathways) in our survival assay. Contrary to the pronounced effect observed in sek-1; glp-4 nematodes, we found that the response of glp-4 nematodes to Harmane mirrored the minor response of the daf-16; glp-1 nematodes (Figure 5). There was only a one day extension of lifespan, as a result of Harmane. From this result we concluded that Harmane does not affect the insulin-like receptor pathway.

### Harmane induces the immune response gene F35E12.5

We wondered how it could be that Harmane had a much more pronounced effect in the sek-1; glp-4 mutant, than in the glp-4 nematode or the daf-16; glp-1 mutant. One explanation could be that Harmane activates the p38 MAPK pathway downstream of the SEK-1 protein. We did not consider this as a possibility, since this effect would probably also be seen in the glp-4 worms and the daf-16; glp-1 mutant. Also, it was recently reported by Pukkila-Worley et al. that activation of the p38 MAPK pathway, by the small molecule RPW-24, resulted in a marked reduction in intestinal colonization by P. aeruginosa [4]. We did not observe such a decrease, in colonization with E. coli EDL933, with Harmane (Figure 3).

However, we decided to test the effect of Harmane on F35E12.5. This protein is a putative immune effector, and it has been found to be strongly induced during infection with P. aeruginosa [6] and Y. pestis, but not when fed on E. coli OP50 [7]. Troemel et al. and Bolz et al. found that this strong inducible expression of F35E12.5 was mediated by the p38 MAPK immune pathway. However, they also found weak inducible expression of F35E12.5 in pmk-1 mutant nematodes (these are defective in the p38 MAPK pathway, similar to sek-1 mutants). Again this induction was only seen on P. aeruginosa and Y. pestis, not on E. coli OP50. They proposed the existence of an immune pathway parallel to the p38 MAPK pathway.

**Figure 6:**
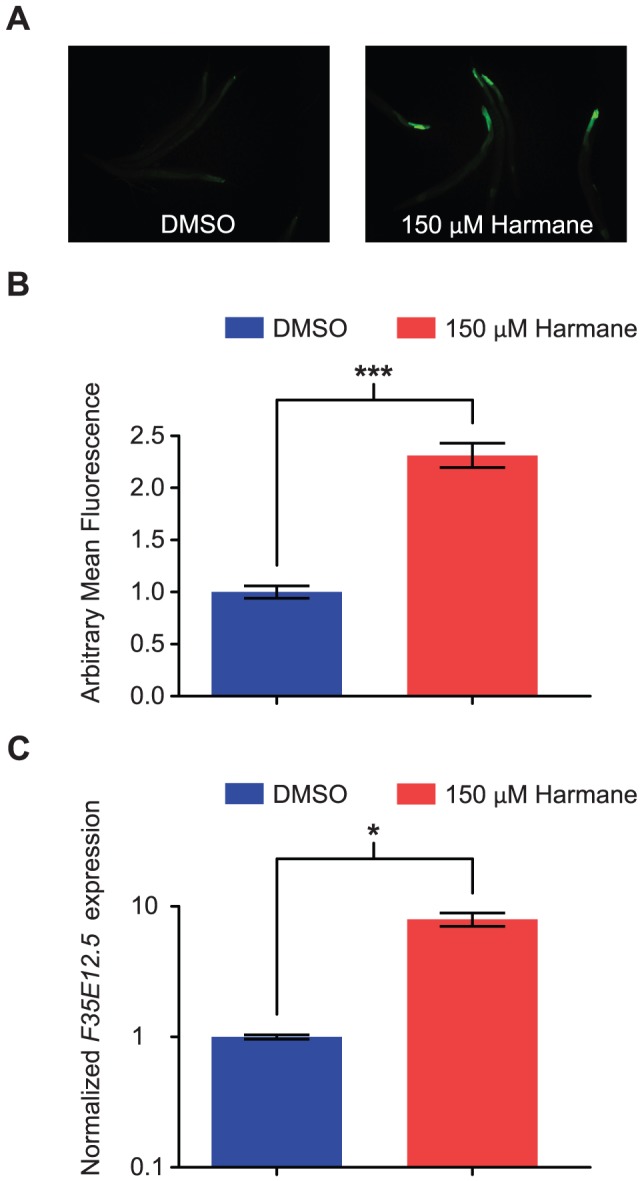
Harmane induces the immune response gene *F35E12.5*. (A) Fluorescence microscopy pictures of *F35E12.5::gfp* transgenic *C. elegans*, exposed to DMSO or Harmane for 20 hours, at 15°C (feeding on *E. coli* OP50). The fluorescence was always seen at the tail end of the animals. (B) Individual nematodes in fluorescence pictures were quantified, and data normalized to the level of the DMSO treated nematodes. There was a significant difference between the two samples [*P*<0.0001; DMSO, n = 54; 150 µM Harmane, n = 54; error bars indicate SEM]. (C) Verification of *F35E12.5* induction by Harmane by qRT-PCR. Bar graphs represent relative expression levels normalized to the control treatment (DMSO). 20 hours treatment with Harmane resulted in a significant induction (*P* = 0.0175) of *F35E12.5*. Error bars indicate SEM derived from two independent biological replicates.

To determine whether Harmane could induce F35E12.5, we exposed a wild-type C. elegans strain carrying a F35E12.5::gfp transgene [7] to Harmane or DMSO, for 20 hours. We analyzed the worms by fluorescence microscopy, and quantified the expression of F35E12.5::gfp (Figure 6A and 6B). We found a significantly stronger expression in the transgenic nematodes treated with Harmane (P<0.0001). This result was confirmed by qRT-PCR analysis on wild-type Bristol N2 nematodes, after 20 hours treatment with Harmane or DMSO. F35E12.5 was significantly up-regulated in response to Harmane (P = 0.0175). Thus, Harmane exhibits immune-inductive activity. However the up-regulation of F35E12.5 by Harmane in AY101 (F35E12.5::gfp) and Bristol N2 nematodes were many fold less than the up-regulation observed by Troemel et al. and Bolz et al. in response to P.aeruginosa and Y. pestis. This indicates that the p38 MAPK pathway is not the main target of Harmane.

Instead we propose the hypothesis that Harmane stimulates more than one pathway involved in pathogen resistance and longevity. This stimulon may include the p38 MAPK pathway. However, based on our results showing that Harmane has a much more pronounced effect in the sek-1; glp-4 mutant, than in the glp-4 or daf-16; glp-1 mutants, we hypothesize that Harmane targets one or more alternative immune responses and that these are being up-regulated in the absence of the p38 MAPK pathway. This phenomenon of up-regulation of immune effector genes in order to compensate for loss of genes encoding similar effectors has recently been reported in C. elegans [20].

## Conclusion

We have shown here that the alkaloid compound Harmane increases the lifespan of nematodes infected with a human pathogen, the Shiga toxin-producing *Escherichia coli* EDL933 and several other bacterial pathogens. This was shown to be unrelated to the weak antibiotic effect of Harmane. We found that the effect of Harmane was more pronounced in worms deficient in the p38 MAPK pathway (*sek-1*; *glp-4* mutants). Worms deficient in the insulin-like receptor pathway (*daf-16*; *glp-1* mutants) experienced the same minor lifespan extension as *glp-4* worms. We then demonstrated that Harmane induces the immune effector gene *F35E12.5*. This leads us to believe that Harmane stimulates the innate immune response of the nematode. We hypothesize that at least part of this stimulon involves other constituents than p38 MAPK and insulin-like signaling, however, these constituents and their activation by Harmane remains to be elucidated. Interestingly, the activity of Harmane did not lower the overall colonization-burden of live bacteria, despite rescuing the nematodes. Also, Harmane treatment increased the lifespan of worms feeding on non-viable bacteria. We therefore hypothesize that the response induced by Harmane is likely involved in general stress management rather than a direct antimicrobial response against pathogens. We believe that Harmane could be used as a tool to further investigate the complexity of the innate immune system of *C. elegans*; together with other immune-stimulatory drugs [4].

It may seem strange that an organism like *C. elegans*, which has evolved alongside numerous microorganisms, would benefit from outside drug intervention, targeting host innate immunity. However, *C. elegans* is not likely to meet many human pathogens in its natural environment (in the soil). It is conceivable that many of these pathogens do not trigger the innate immune system of the nematode. This is similar to the situation in humans, where for example *E. coli* is usually a commensal inhabitant of the large intestine. Immunomodulating drugs have therefore been proposed as a possible alternative to classic antibiotics [21]. Since the innate immune system of *C. elegans* has a high degree of evolutionary conservation, it is conceivable that results from the worm model could be extended to higher organisms, including humans. *C. elegans* could therefore prove useful for selection and development of such compounds. The immuno-stimulatory effect observed for Harmane could provide a scaffold upon which further elaborations on this paradigm are possible.

## Materials and Methods

### C. elegans and bacterial strains used

The Caenorhabditis elegans strains used in this study were: C. elegans N2 Bristol [22], C. elegans AU37 [sek-1(km4); glp-4(bn2) I] (MAPK kinase deficiency and temperature-sensitive sterile) [3], C. elegans AU147 [daf-16(mgDf47) I; glp-1(e2141) III] (transcription factor DAF-16 deficiency and temperature-sensitive sterile), C. elegans SS104 [glp-4(bn2) I] (temperature-sensitive sterile), C. elegans TJ356 [zIs356 [daf-16::gfp+ rol-6(su1006)] [19] and C. elegans AY101 [F35E12.5p::gfp+rol-6(su1006)] [7]. All C. elegans strains were maintained and propagated on NGM media [23], with E. coli OP50 as food source.

The bacterial strains used were: Escherichia coli OP50, E. coli O157:H7 strain EDL933 [24] (Shiga toxin-producing E. coli strain), Salmonella Typhimurium strain C17 [12], [13], Pseudomonas aeruginosa strain PA14 [14] and Enterococcus faecalis strain OG1RF [3], [15]. Bacterial strain were grown in LB media at 37°C, except E. faecalis which was grown in brain hearth infusion media (BHI) (OXOID Ltd.) at 37°C.

### C. elegans bacterial infection assays

A synchronous population of nematodes was obtained, by releasing worm embryos using alkaline hypochlorite treatment [23]; followed by hatching of the eggs and L1 arrest in M9 buffer at 15°C overnight. Synchronous L1 larvae were transferred to nematode growth medium (NGM) agar plates [23] seeded with OP50 and allowed to develop into sterile adult/L4 larvae, by incubating for two days at 25°C. Hereafter they were washed three times in M9 buffer and transferred to assay plates, with bacterial lawns. Assay plates were prepared on 60-mm culture plates with NGM agar supplemented with 0.3% DMSO or the indicated concentrations of Harmane (Sigma-Aldrich, CAS Number 486-84-0). Exceptions to this were the plates for Enterococcus faecalis, which were BHI agar (OXOID Ltd., supplemented with 5 mg/ml cholesterol) and the plates for heat-killed E. coli EDL933, which were NGM agar supplemented with 25 µg/ml of chloramphenicol (in order to inhibit any viable E. coli EDL933 or residual E. coli OP50).

The plates were seeded with 20 μl of overnight culture, of bacterial strains, followed by overnight incubation at 37°C. After acclimatization of the plates to room temperature, about 50 worms were transferred to each plate. Plates were incubated at 25°C and scored for dead worms each day. A worm was considered dead when it failed to respond to a touch with a platinum wire; dead worms stuck to the wall of the plate were censored from analysis. Survival data from duplicate or triplicate plates were pooled and subjected to survival analysis. Results presented are representative of repeated independent assays.

### Growth assays with E. coli EDL933 subjected to Harmane or tetracycline

The growth kinetics of E. coli EDL933 in the presence of varying concentrations of Harmane or the antibiotic tetracycline was determined in NGM medium. An overnight culture of E. coli EDL933 was diluted into fresh NGM medium, supplemented with 0.3% DMSO or Harmane at the concentrations: 50 µM and 150 µM. Tetracycline was tested at a concentration of 0.1 µg/ml. All cultures were prepared in triplicate. The cultures were incubated at 37°C, with rigorous shaking and samples were taken at 4, 8 and 24 hours. Samples were serially diluted and 5 µl of each dilution spotted on an LB-agar plate. The plate was incubated overnight at 37°C.

### Visualization and quantification of bacterial intestinal colonization

Synchronised adult/L4 stage C. elegans AU37 (sek-1; glp-4) were transferred from lawns of E. coli OP50 to lawns of E. coli EDL933 carrying pBAD18-GFP, on NGM plates containing 100 µg/ml ampicillin and 0.2% arabinose (in order to activate expression of GFP from the P_BAD_ promoter) and either 0.3% DMSO or 150 µM Harmane. On day 4 the worms were washed from the plates into M9 media followed by 3 further washes to remove external bacteria. Subsequently, the worms were anaesthetized and immobilized, by addition of 1% NaN_3_, and placed on top of a 1.5% agarose pad on a microscope slide. Worms were examined and photographed with an Olympus BX61 microscope and an Olympus DP71 camera using the cel ˆP software (Olympus). All photographs were acquired using the same settings and a fixed exposure time of 50 ms. Quantitative assessment of bacterial colonization was done by determination of fluorescence intensity of nematodes subjected to 0.3% DMSO or 150 µM Harmane, by analysis of individual nematodes, using ImageJ v1.45.

### Analysis of pharyngeal pumping activity

About 30 synchronised L4/young adult C. elegans AU37 (sek-1; glp-4) nematodes were transferred to lawns of E. coli OP50, on NGM plates with either 0.3% DMSO or 150 µM Harmane. After 24 and 48 hours incubation at 25°C, the worm’s pharyngeal grinder activity was measured. We observed the grinder activity of a single adult for 20 sec, using an Olympus SZX7 stereomicroscope. We counted the number of contractions of the terminal bulb, of the grinder. This was done for 10 nematodes on each plate, each day.

### Measuring body length of C. elegans

Synchronised adult/L4 stage C. elegans AU37 (sek-1; glp-4) were transferred to E. coli OP50 lawns, on NGM plates containing either 0.3% DMSO or 150 µM Harmane. On day 1 and 4 the worms were washed from plates into M9 media followed by a single wash to remove external bacteria. The worms were then anaesthetized and immobilized, by addition of 1% NaN_3_, and placed on top of a 1.5% agarose pad on a microscope slide. Worms were examined and photographed with an Olympus SZX7 stereomicroscope and an Olympus SC30 camera, using the Analysis getIT software (Olympus). The length of the worms was measured using ImageJ v1.45, as described in [25]. The sample sizes are given in the legend to figure 4.

### DAF-16 translocation assay

Synchronised adult/L4 stage C. elegans TJ356 (daf-16::gfp) were transferred to lawns of E. coli OP50 on plates with either 0.3% DMSO or 150 µM Harmane. The plates were incubated at 15°C for 1 hour or 20 hours. Then the worms were washed from the plates into M9 media followed by a wash to remove external bacteria. Subsequently, the worms were anaesthetized and immobilized, by addition of 1% NaN_3_, and placed on top of a 1.5% agarose pad on a microscope slide. Worms were examined and photographed with an Olympus BX61 microscope and an Olympus DP71 camera using the cel ˆP software (Olympus). In each experiment (1-hour or 20-hour) we placed one of the DMSO plates at 37°C for 30 minutes, just prior to the nematodes being analysed. This served as a positive control of DAF-16 translocation to the nucleus. We compared the nematodes treated with Harmane to the positive control to look for signs of DAF-16 translocation.

### F35E12.5-GFP visualization and quantification

Synchronised adult/L4 stage C. elegans AY101 (F35E12.5::gfp) were transferred to lawns of E. coli OP50 on plates with either 0.3% DMSO or 150 µM Harmane. The plates were incubated at 15°C for 20 hours. The worms were then washed from the plates into M9 media followed by a wash to remove external bacteria. The worms were anaesthetized and immobilized, by addition of 1% NaN_3_, and placed on top of a 1.5% agarose pad on a microscope slide. Worms were examined and photographed with an Olympus BX61 microscope and an Olympus DP71 camera using the cel ˆP software (Olympus). All photographs were acquired using the same settings and a fixed exposure time. Quantitative assessment of GFP expression was done by determination of fluorescence intensity of nematodes subjected to DMSO or 150 µM Harmane, by analysis of individual nematodes, using ImageJ v1.45.

### Quantitative RT-PCR

Synchronised Bristol N2 nematodes (100–200 animals) fed on OP50 on plates with Harmane or solvent (DMSO) for 20 hours were washed off the plates and transferred into RLT Plus Buffer (Qiagen). Sterile RNase-free bashing beads were added, followed by lysis and homogenization in a TissueLyser II (Qiagen) for 5 min. RNA extraction was hereafter performed with RNeasy Plus Mini Kit (Qiagen) combined with on-column DNase treatment (RNase-Free DNase Set, Qiagen). cDNA was synthesized by the SuperScript III First-Strand Synthesis SuperMix (Invitrogen). Relative expression levels of F35E12.5 was determined and normalized to pan-actin (act-1, -3, -4) using the QuantiTect SYBR Green PCR Kit (Qiagen), a Stratagene MX3000P qPCR machine, and previously published primers [7].

### Statistics

Differences in the survival of C. elegans, in the infection assays, were determined using GraphPad Prism version 5.00 (www.graphpad.com). The Kaplan-Meier method was used to calculate survival fractions and log-rank test was used to compare survival curves. Mean fluorescence of worms feeding on GFP expressing E. coli EDL933 were compared by unpaired, two-tailed Student´s t-test, using GraphPad Prism. Data for ‘pharyngeal pump activity’and ‘body length’ were also analysed by unpaired, two-tailed Student´s t-test in GraphPad Prism. Gene expression fold change from qRT-PCR analysis was analysed using unpaired, two-tailed Student´s t-test in GraphPad Prism. Sample sizes for the different assays are given in the figure legends. Values of P≤0.05 were considered statistically significant.

## Supporting Information

Figure S1 The Intimin and Tir interaction only plays a minor role in pathogenicity of E. coli EDL933 towards C. elegans. Harmane strongly extends lifespan.
(PDF)

Figure S2 C. elegans AU37 nematodes show no avoidance behavior against Harmane.
(PDF)

Table S1 Minimum inhibitory concentrations of tetracycline and Harmane in NGM media towards E. coli EDL933, S. Typhimurium C17, P. aeruginosa PA14 and E. faecalis OG1RF.
(PDF)

Method S1 Two-hybrid screen for inhibitors of the Intimin and Tir (translocated intimin receptor) interaction.
(PDF)

Method S2 Determination of minimum inhibitory concentration.
(PDF)

Method S3 Avoidance assay for C. elegans.
(PDF)
