# Supplementary material for: The Alkaloid Compound Harmane Increases the Lifespan of Caenorhabditis elegans during Bacterial Infection, by Modulating the Nematode’s Innate Immune Response
Source: PLoS One. 2013 Mar 27;8(3):e60519. doi: 10.1371/journal.pone.0060519 (PMC3609739; doi:10.1371/journal.pone.0060519)
Supplement: Figure S1 — The Intimin and Tir interaction only plays a minor role in pathogenicity of E. coli EDL933 towards C. elegans. Harmane strongly extends lifespan. (PDF) [file pone.0060519.s001.pdf]

Figure S1

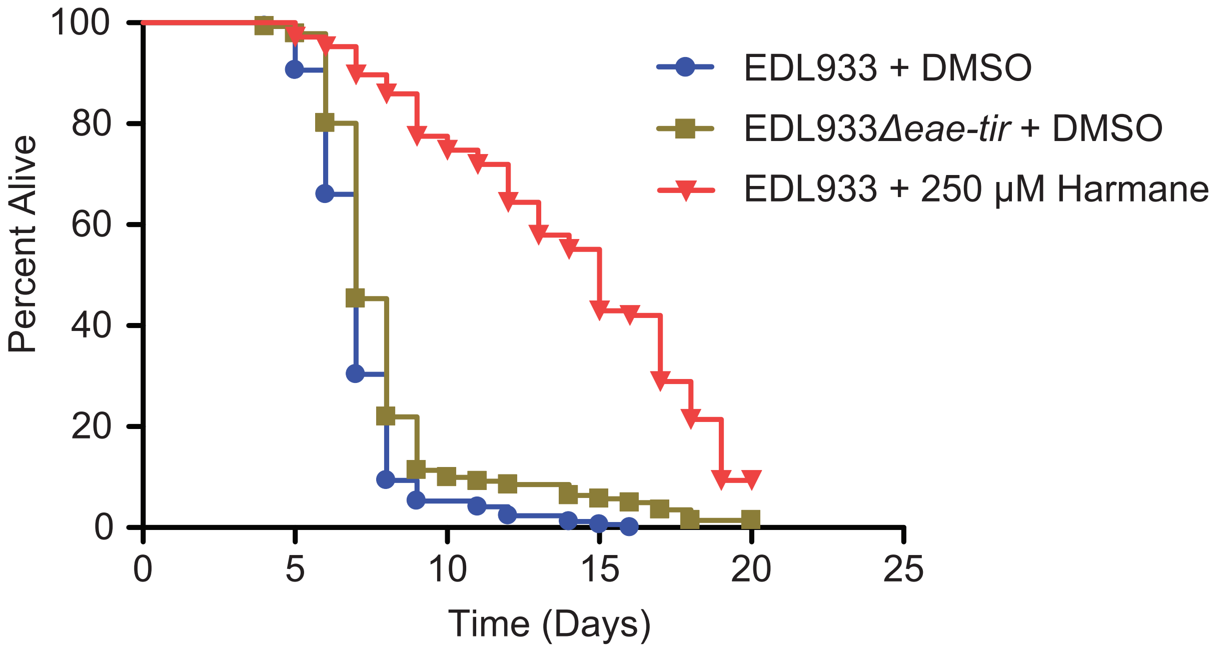

The Intimin and Tir interaction only plays a minor role in pathogenicity of *E. coli* EDL933 towards *C. elegans*. Harmane strongly extends lifespan. Infection assay on *C. elegans* AU37 (*sek-1*; *glp-4*) nematodes with wild-type *E. coli* EDL933 grown on plates with 0.3% DMSO; *E. coli* EDL933Δ*eae-tir* (Lacking Intimin and Tir) grown on plates with 0.3% DMSO and *E. coli* EDL933 grown on plates with 250 µM Harmane. The curve for *E. coli* EDL933Δ*eae-tir* is significantly different ($P<0.001$) from the wild-type, but the median survival of nematodes was 7 days on both. The median survival of nematodes on 250 µM Harmane was 15 days. [Number of animals: *E. coli* EDL933 + DMSO, n=171; *E. coli* EDL933Δ*eae-tir* + DMSO, n=139; *E. coli* EDL933 + 250 µM Harmane, n=97].
