# Supplementary material for: The Alkaloid Compound Harmane Increases the Lifespan of Caenorhabditis elegans during Bacterial Infection, by Modulating the Nematode’s Innate Immune Response
Source: PLoS One. 2013 Mar 27;8(3):e60519. doi: 10.1371/journal.pone.0060519 (PMC3609739; doi:10.1371/journal.pone.0060519)
Supplement: Figure S2 — C. elegans AU37 nematodes show no avoidance behavior against Harmane. (PDF) [file pone.0060519.s002.pdf]

Figure S2

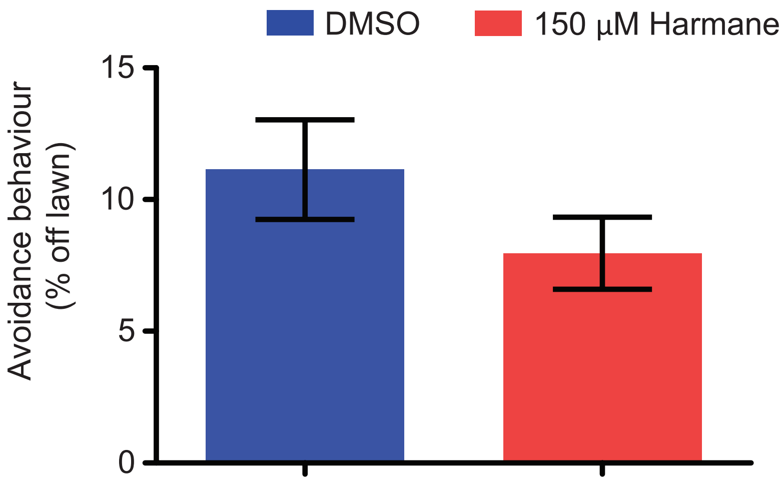

***C. elegans* AU37 nematodes show no avoidance behavior against Harmane.** The average percentage of worms that were outside the bacterial lawn of *E. coli* OP50, supplemented with either DMSO or Harmane, after 16 hours of exposure. There was no significant difference between the two samples. [DMSO, n=365; 150 µM Harmane, n=644; error bars indicate SEM].
