# Supplementary material for: The Alkaloid Compound Harmane Increases the Lifespan of Caenorhabditis elegans during Bacterial Infection, by Modulating the Nematode’s Innate Immune Response
Source: PLoS One. 2013 Mar 27;8(3):e60519. doi: 10.1371/journal.pone.0060519 (PMC3609739; doi:10.1371/journal.pone.0060519)
Supplement: Table S1 — Minimum inhibitory concentrations of tetracycline and Harmane in NGM media towards E. coli EDL933, S. Typhimurium C17, P. aeruginosa PA14 and E. faecalis OG1RF. (PDF) [file pone.0060519.s003.pdf]

Table S1: Minimum inhibitory concentrations of tetracycline and Harmane in NGM media towards *E. coli* EDL933, *S.* Typhimurium C17, *P. aeruginosa* PA14 and *E. faecalis* OG1RF.

Strain	Growth	No Growth
E. coli EDL933	0.31 µg/ml Tetracycline	0.62 µg/ml Tetracycline
E. coli EDL933	0.25 mg/ml (1372 µM) Harmane	0.5 mg/ml (2744 µM) Harmane
S. Typhimurium C17	0.125 mg/ml (686 µM) Harmane	0.25 mg/ml (1372 µM) Harmane
P. aeruginosa PA14	0.5 mg/ml (2744 µM) Harmane	1 mg/ml (5488 µM) Harmane
E. faecalis OG1RF	0.5 mg/ml (2744 µM) Harmane	1 mg/ml (5488 µM) Harmane
