# Supplementary material for: The Alkaloid Compound Harmane Increases the Lifespan of Caenorhabditis elegans during Bacterial Infection, by Modulating the Nematode’s Innate Immune Response
Source: PLoS One. 2013 Mar 27;8(3):e60519. doi: 10.1371/journal.pone.0060519 (PMC3609739; doi:10.1371/journal.pone.0060519)
Supplement: Method S1 — Two-hybrid screen for inhibitors of the Intimin and Tir (translocated intimin receptor) interaction. (PDF) [file pone.0060519.s004.pdf]

Method S1: Two-hybrid screen for inhibitors of the Intimin and Tir (translocated intimin receptor) interaction.

Harmane was identified in a two-hybrid screen for inhibitors of the interaction between the enterohemorrhagic *E. coli* virulence factors, Tir and Intimin. The two-hybrid screen is based on the method devised by Karimova et al [1]where a stable interaction between the fusion subunits produces an active adenylate cyclase which activates transcription of the *lac* operon. We constructed plasmids pKT25-Tir and pUT18-Intimin using the full length coding sequence for each and strain BTH101 was transformed with these plasmids. The two vector plasmids, and pKT25-zip and pUT18-zip (containing a leucine zipper motif) in BTH101, served as the negative and positive controls respectively. The plasmid-containing strains were arrayed in 96 well plates in 200 µl of Davis and Mingioli minimal medium [2] supplemented with 0.2% maltose, ampicillin (50 µg/ml), kanamycin (25 µg/ml), thiamine (2 µg/ml), X-Gal (40 µg/ml) and IPTG (0.5 mM). The plates were incubated at 30°C and scanned at a wavelength of 664 nm after 48 and 72 hr on a Tecan Safire plate reader. Relative to the positive control, Harmane (10 µM) reduced the absorbance by 90% in BTH101 with the recombinant plasmids but had no effect on BTH101 with the leucine zipper control plasmids. Harmane for the screen was supplied by the University of Massachusetts Medical School Small Molecule Screening Core Facility which also performed the plate reading and data analysis.

1. Karimova G, Pidoux J, Ullmann A, Ladant D (1998) A bacterial two-hybrid system based on a reconstituted signal transduction pathway. Proc Natl Acad Sci U S A 95: 5752-5756.

2. Davis BD, Mingioli ES (1950) Mutants of *Escherichia coli* requiring methionine or vitamin B12. J Bacteriol 60: 17-28.
