# Supplementary material for: The Alkaloid Compound Harmane Increases the Lifespan of Caenorhabditis elegans during Bacterial Infection, by Modulating the Nematode’s Innate Immune Response
Source: PLoS One. 2013 Mar 27;8(3):e60519. doi: 10.1371/journal.pone.0060519 (PMC3609739; doi:10.1371/journal.pone.0060519)
Supplement: Method S2 — Determination of minimum inhibitory concentration. (PDF) [file pone.0060519.s005.pdf]

Method S2: Determination of minimum inhibitory concentration

The minimum inhibitory concentrations of Harmane and tetracycline were performed in analogy to standard broth microdilution procedures, with slight modifications. Test strains were inoculated (at approximately $5*10^5$ CFU/ml) into Nematode Growth Medium containing two-fold dilutions of Harmane or tetracycline followed by incubation at 37°C for 24 hours. *E. faecalis* was cultured under the same conditions, but in brain hearth infusion media (BHI) (OXOID). The lowest concentration preventing visual growth was noted.
