# Supplementary material for: The Alkaloid Compound Harmane Increases the Lifespan of Caenorhabditis elegans during Bacterial Infection, by Modulating the Nematode’s Innate Immune Response
Source: PLoS One. 2013 Mar 27;8(3):e60519. doi: 10.1371/journal.pone.0060519 (PMC3609739; doi:10.1371/journal.pone.0060519)
Supplement: Method S3 — Avoidance assay for C. elegans. (PDF) [file pone.0060519.s006.pdf]

Method S3: Avoidance assay for *C. elegans*.

C. elegans AU37 aversion behavior towards Harmane was evaluated essentially as previously described [1]. NGM plates seeded with a lawn of OP50 were instilled with 28 µl of 50 mM Harmane (final concentration 150 µM Harmane) or an equal volume of DMSO onto the center of the lawn and allowed to dry (1 hour at room-temperature). Hereafter, 50-100 synchronized adult animals were added to the center of the lawn and animals were scored as either on or off the lawn after 16 hours of incubation at 25°C. Data represent mean aversion behavior from six plates per condition and error bars correspond to SEM

1. Pukkila-Worley R, Feinbaum R, Kirienko NV, Larkins-Ford J, Conery AL, et al. (2012) Stimulation of Host Immune Defenses by a Small Molecule Protects *C. elegans* from Bacterial Infection. PLoS Genet 8: e1002733.
